# Assessment and forecasting of particulate matter emissions and structural health monitoring of buildings in Bangalore

**DOI:** 10.1038/s41598-025-00814-9

**Published:** 2025-05-22

**Authors:** L. Pinky Devi, R. Chandana, Din Bandhu

**Affiliations:** 1 Department of Civil Engineering, Manipal Institute of Technology Bengaluru, Manipal Academy of Higher Education, Manipal, 576104 Karnataka India; 2 Department of Civil Engineering, Nagarjuna College of Engineering and Technology, Bengaluru, 562110 Karnataka India; 3 Independent Researcher, Bengaluru, 560064 Karnataka India

**Keywords:** Particulate matter, Emissions, AQI, Regression modeling, Structural health monitoring, Environmental sciences, Mathematics and computing

## Abstract

Particulate Matter (PM) emissions have emerged as a critical global concern due to rapid urbanisation, increased vehicular traffic, and construction activities. These emissions not only harm human health and the environment but also degrade building materials, posing a threat to infrastructure. This study focuses on assessing PM emissions, forecasting Air Quality Index (AQI) levels, and evaluating the structural health of buildings in Bangalore. Data from 12 monitoring stations across the city, collected between 2013 and 2021, were analysed to identify key pollutants, seasonal variations, and their impact on buildings. The study reveals that PM_10_ and PM_2.5_ are the primary pollutants, with concentrations peaking during summer and winter, while monsoon seasons show lower levels. A forecasting model with 93% accuracy was developed to predict AQI levels, demonstrating a strong correlation between predicted and actual values. Structural health monitoring, conducted using Non-Destructive Testing methods, highlights significant deterioration in buildings located in high-pollution areas, such as the Peenya Industry and K.R. Market. The findings underscore the urgent need for measures to mitigate pollution’s impact on both public health and infrastructure. This study provides valuable insights for policymakers and urban planners to develop targeted strategies for improving air quality and preserving building integrity in rapidly urbanising cities.

## Introduction

As urbanisation continues to draw more people into cities worldwide, the concentration of particulate matter (PM) in urban air quality emerges as a significant global concern. Poor air quality poses a health risk to everyone and contributes substantially to climate change by exacerbating global warming. Building operations play a substantial role in air pollution, accounting for 50% of climate change and 23% of all air pollution^1^. Although building activities contribute only 4% to total PM emissions in urban areas, traffic-related emissions represent over 50% of these emissions. Currently, more than 56.6% of the global population residing in metropolitan areas is exposed to harmful levels of air pollution^2^. Exposure to high concentrations of ambient PM is linked to approximately 40% of respiratory diseases, contributing to over 1.5 million deaths worldwide annually.

The primary sources of outdoor PM concentrations in metropolitan areas include waste incineration, construction activities, emissions from tyre and brake wear, and road dust re-suspension. India has recently witnessed unprecedented economic growth and rapid urbanisation, leading to urban expansion, an increase in urban population density, a rise in vehicular traffic, severe traffic congestion, extensive construction activities, and temporary land use changes. Air pollution refers to human-made pollutants released into the atmosphere, which have detrimental effects on both human health and the environment. According to the World Health Organization^3^, approximately 6.8 million people die prematurely each year due to poor air quality. These pollutants come in various forms, including gas molecules, solid particles, or liquid droplets. The primary types of pollution-causing pollutants present in the air contribute to a range of harmful effects. These include respiratory and heart diseases, health issues in children, exacerbation of global warming, acid rain formation, eutrophication of water bodies, adverse impacts on wildlife, and depletion of the ozone layer^4^.

The Air Quality Index (AQI) serves as a vital tool for assessing the cleanliness or pollution levels in the air. Like a thermometer’s scale ranging from 0 to 500 °C, the AQI provides a measure of air quality, but unlike temperature, it reflects changes in pollution concentration rather than temperature variations. Government agencies utilise the AQI to inform the public about current or projected air pollution levels, with higher AQI values indicating increased risks to public health^5^. The Environmental Protection Agency (EPA) identifies six pollutants, known as “criteria” air pollutants, and establishes and regulates acceptable levels for human health. These pollutants include sulfur oxides, lead, surface ozone, nitrogen oxides, carbon monoxide, and particulate matter. By setting standards for these pollutants, the EPA aims to manage and mitigate their impact on public health and the environment.

Particulate Matter (PM) is a complex mixture of solid particles and liquid droplets suspended in the air, containing various components such as sulfates, nitrates, ammonia, sodium chloride, black carbon, mineral dust, and water. NOx, which primarily appears as a brown gas, is largely generated by vehicles such as automobiles, trucks, and land vehicles (including boats and construction equipment), as well as industrial sources like power plants, industrial boilers, cement kilns, and turbines. PM2.5 refers to airborne particulate matter with a diameter of 2.5 µm or less, formed as a result of atmospheric chemical reactions and fuel combustion. Major contributors to PM2.5 emissions include automobiles, power plants, industrial facilities, residential sources like home fireplaces and wood stoves, agricultural activities, and wildfires. On the other hand, PM10 encompasses a broader range of particles, including smoke, dust, salt, acids, metals, and other airborne particles smaller than 10 µm in diameter. PM10 consists of coarse PM composed of suspended solids or liquids. PM10 can originate from various sources and activities, including industrial processes, vehicle emissions, construction activities, and natural sources like dust storms^6^.

National Ambient Air Quality Standards (NAAQS) are air quality guidelines set by regulatory bodies like the Central Pollution Control Board (CPCB) at the national level. The NAAQS establishes limits for the concentration of six air pollutants, including those contributing to smog, acid rain, and posing health hazards. These standards are crucial for maintaining and improving air quality, thereby safeguarding public health and the environment. Air quality standards apply primarily to outdoor air and measure the levels of pollutants in the air.

The Air Quality Index (AQI) simplifies the complex data regarding the six main pollutants that impact air quality into a single numerical value, along with corresponding terms and colours indicating the severity of air pollution. The National Air Quality Index was launched on October 17, 2014, aiming to offer the public straightforward information about the current state of air quality. This index serves as a crucial tool for individuals and authorities to understand and respond to air pollution levels, helping to protect public health and the environment.

An examination and explanation of the daily, weekly, and seasonal patterns of hourly average concentrations of particulate matter (PM10, PM2.5, and PM1) near the Chennai urban expressway found that PM data unveils distinct daily, weekly, and seasonal fluctuations at the research site^4^. During the daily cycle, PM concentration peaks during weekday rush hours and decreases during afternoon and nighttime hours. Seasonally, PM concentrations are highest during the post-monsoon period compared to winter and summer seasons. Analysis of 24 h average concentrations of PM10 and PM2.5 indicated peak values during monsoons and winter and minimum values during summer resulting from varying traffic conditions in Chennai city during monsoon, winter, and summer seasons study^7^. Furthermore, a positive matrix decomposition of PM10 and PM2.5 emission sources along urban roads in Chennai City results demonstrate that the 24-h average concentration of PM10 was notably higher in winter and monsoon seasons compared to summer. In contrast, PM2.5 concentration exhibited a slight increase during monsoon and summer^8^. A study focusing on PM emissions found that the mass concentration of PM10 and PM2.5 resulted from varying traffic conditions in Chennai city. Chemical characterisation of PM10 and PM2.5 samples collected across 22 instances for each season revealed a prevalence of crustal elements such as Ca, Mg, Al, Fe, and K^6^. A review to evaluate the impact of road traffic emissions on PM concentrations through field measurements underscores the substantial contribution of transport emissions to primary particle emissions within urban environments. However, it also draws attention to the limited quantitative understanding of their contribution to particle concentrations, particularly concerning non-emission sources^9^. The study focused on assessing the influence of road transport emissions on fine particulate matter (PM2.5) concentrations in London during the year 2008, utilising the OSCAR Air Quality Rating System^10^. Unlike prior studies, this research focused solely on PM2.5 mass, employing distinct methodologies. Modelling results indicate that approximately two-thirds of the traffic-related increase stems from emissions, while the remaining portion is primarily attributed to wear from brakes and tyres.

In specific industrial and managed regions of India, the impact of air pollutants on human health and vegetation resulted in a notable increase in hospitalisations during the winter period^11^. Analysis of lung function conducted in both study areas revealed that individuals living in industrial zones demonstrated lower air exchange rates in comparison to those residing in control areas. In another study, the hourly average concentrations of PM2.5 and NO2 at a busy intersection in Delhi and on a bustling road in Chennai are evaluated^12^. They introduced the best-fitting statistical distribution model (SDM) for each city. The findings demonstrated that lognormal and logistic distribution models optimally described NO2 concentrations during the winter and summer seasons. Furthermore, a systematic approach for developing hybrid models to predict hourly average and percentile ranges of PM2.5 and NOx concentrations in urban settings, specifically Delhi and Chennai^13^. Sunil et al.^13^ study investigates the characteristics of PM mass concentrations (PM10 and PM2.5) and their correlation with meteorological conditions in Pune city from 2011 to 2012. Analysis of 24-h average PM10 and PM2.5 concentrations reveals peak levels during winter, followed by summer and post-monsoon seasons^14^. A study was conducted to project global primary particulate matter (PM) emissions originating from road vehicle activities spanning from 2010 to 2050. This forecast is based on four widely adopted global fuel economy scenarios and relies on SPEW-Trend, a dynamic vehicle population model grounded in exhaust gas characteristics. Unlike previous global emissions models, this approach integrates detailed information on the technology inventory, including vehicle types, years, and quantities of highly emitting sources^15^. The study investigates the impact of air pollution from brick kilns on plant species in Bangladesh and evaluates their potential for pollution mitigation. The findings support the integration of these species into urban planning to improve air quality and mitigate pollution from brick kilns and other industrial sources^16^. Furthermore, it also explores the effects of aerosol optical properties on Direct Aerosol Radiative Forcing (DARF) in four distinct climatic zones. The findings indicate that changes in aerosol optical depth play a significant role in local atmospheric warming or cooling, with scattering aerosols pointing toward a potential cooling effect. The study highlights the importance of region-specific aerosol analysis to enhance climate modelling and inform policy decisions^17^.

Spiru et al.^18^ directed their focus toward assessing the impacts of exposure to varying pollution levels on human health. They aimed to comprehend how pollutants, escalating urban population density, the utilisation of new synthetic materials, and traffic emissions synergistically contribute to exacerbating negative human health effects and deteriorating indoor air quality^18^. Watt et al.^19^ examined the primary environmental repercussions of pollution-induced heritage degradation management, along with the chief characteristics and causes of intensified air pollutants. Sulfur dioxide (SO2) and nitrogen oxides (NOx) emitted from power plants and other sources combine with moisture in humid climates, forming acids that precipitate and corrode cultural assets and impact human health. A review examining the impact of air pollution and climatic variations on historical monuments in India within a global framework highlights that changes in outdoor air quality are largely driven by traffic congestion and industrial emissions. In contrast, indoor air quality is mainly affected by inadequate ventilation systems^20^.

A study on enhancing the accuracy of low-cost particulate matter (PM) sensors (LCS) in Timisoara, Romania, addresses the limitations of LCS devices, which, while affordable and widely used, often produce biased readings due to factors like aerosol properties and environmental conditions. The study highlights the importance of high spatial and temporal resolution in air quality monitoring to capture localised pollution sources^21^.

Kumar et al.^22^ delved into investigating how air pollution and environmental shifts affect the durability of buildings and transportation structures. Their findings underscored the impact of climate change, pollution, and ecological factors on the integrity of building infrastructure^22^. A detailed analysis is conducted to examine the effects of air pollutants on a wide range of building materials, including stone, brick, mortar, concrete, glass, metals (such as iron, zinc, copper, bronze, aluminium, lead, and silver), polymers, paints, and wood^23^. Additionally, particulate matter, often emitted from diesel vehicles, along with the surface oxidation of sulfur dioxide, can lead to darkening of the material’s surface if soot accumulates.

A comprehensive review of AQI regression modelling projects is conducted to enhance understanding in this field^24–26^. Pallarés et al.^27^ conducted a review of both static and dynamic studies related to Structural Health Monitoring (SHM) and Non-destructive Testing (NDT) techniques in thin masonry structures, discussing the diverse range of strategies employed^27^. Recent advancements in non-destructive testing methods, including scanning frequency, ground radar, infrared technology, fibre optic sensors, and acoustic emission techniques, are carried out^28^. NDT on marine composites to identify various types of defects and a comprehensive overview of available SHM and NDT techniques, along with case studies and investigations into the implementation of SHM in timber structures were carried out^29,30^.

Analysis and evaluation have primarily focused on PM1, PM2.5, and PM10 pollutants, with limited attention given to NOx, SO2, and Lead. The focus of much research has been on heterogeneous traffic emissions near urban areas, with particular attention given to cities like Chennai, Delhi, and Pune. However, there has been limited exploration of monitoring stations indicating elevated pollution levels, particularly in Bangalore. Data collection has primarily relied on Air Quality Monitor instruments, with limited emphasis placed on surveying and expert interviews. Data collection has relied mainly on Air Quality Monitor instruments, with limited emphasis placed on surveying and expert interviews.

Moreover, although the calculation of the Air Quality Index (AQI) is widely applied to pollutants such as PM10, PM2.5, and PM1, there is a significant lack of research on expanding AQI calculations to include a broader range of emissions, such as PM10, PM2.5, NOx, SO2, NH3, and lead. Additionally, research on structural health monitoring, particularly in comparing the effects of pollution on old versus new buildings in outdoor environments, has been notably limited. Also, the Air Quality Index (AQI) serves as a crucial tool for monitoring and communicating air quality levels to the public. It aggregates data on key pollutants, including PM_10_, PM_2.5_, NO_x_, SO_2_, and others, into a single numerical value, providing a simplified measure of air pollution’s severity. However, while AQI is effective for assessing short-term air quality, its long-term implications on infrastructure, particularly building materials, remain underexplored. Prolonged exposure to high levels of pollutants can lead to the deterioration of concrete, corrosion of steel reinforcements, and overall structural weakening, posing significant risks to urban infrastructure.

This study aims to address this gap by focusing on three key objectives:

1. *Identifying and analysing seasonal variations in key pollutants* (PM_10_, PM_2.5_, NO_x_, SO_2_, NH_3_, and Lead) across Bangalore.
2. *Developing a forecasting model* to predict AQI levels, enabling proactive air quality management.
3. *Assessing the impact of air pollution on building structures* through structural health monitoring (SHM) using Non-destructive Testing (NDT) techniques.

By analysing data from 12 monitoring stations across Bangalore from 2013 to 2021, this study provides a comprehensive understanding of pollution trends and their impact on urban infrastructure. The findings highlight the urgent need for integrated strategies to mitigate air pollution’s dual implications on public health and building durability. This research contributes to the growing body of knowledge on urban air quality management. It offers actionable insights for policymakers, urban planners, and environmental engineers to develop sustainable solutions for rapidly urbanising cities.

## Materials and methods

**Fig. 1:**
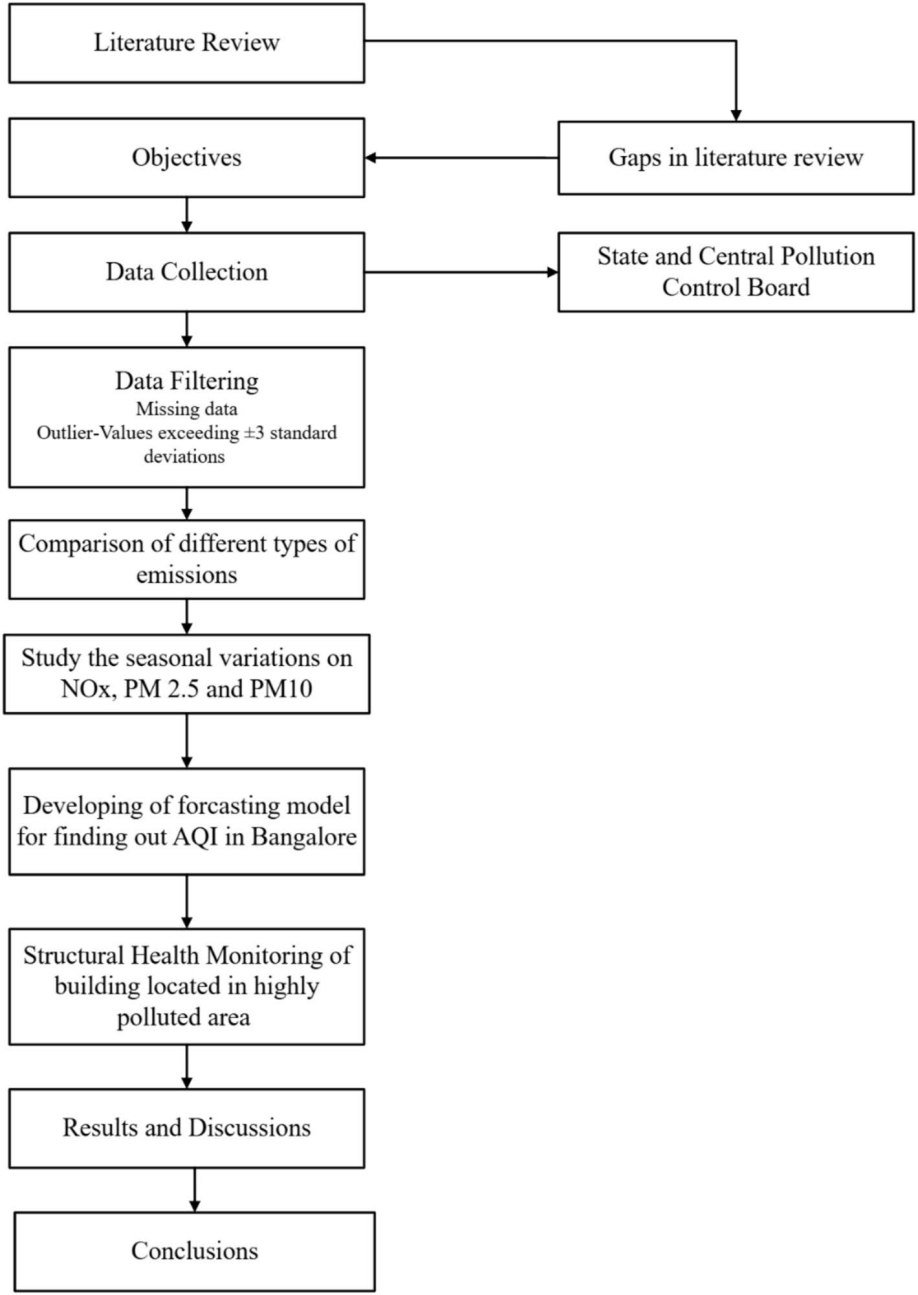
Methodology flowchart for this study.

Figure 1 outlines the methodology adopted in this study, which began with a comprehensive literature review across various categories to deepen the understanding of the project. This review facilitated the identification of key findings and methodologies employed in prior research. Addressing the gaps highlighted in the literature, the project’s objectives and scope were precisely outlined. The subsequent phase focused on data collection, during which ambient air quality data related to various pollutants was obtained from the Karnataka State Pollution Control Board in Bengaluru city. Data was obtained from 12 monitoring stations strategically located across the North, South, West, East, and Central regions of Bangalore, capturing concentrations of pollutants such as SO2, NOx, PM10, PM2.5, NH3, and Lead from 2013 to 2021. The study used data from 12 monitoring stations (2013–2021) with gaps filled via linear interpolation after verifying station logs. Values exceeding ± 3 standard deviations from monthly means were classified as outliers and excluded. The study found that the missing data accounted for < 5% of the dataset. Outliers of the data were less than 1% of the dataset and were omitted from further analysis. After preprocessing, 94% of the original data was retained. The forecasting model used 80% of this cleaned data (2013–2019) for training and 20% (2020–2021) for testing. Following data collection, a comparative analysis of the six pollutants was conducted to identify those with the highest concentrations and to pinpoint the monitoring station recording the most severe pollution levels. This analysis also explored the factors contributing to elevated emissions in specific locations. Subsequently, the study investigated seasonal variations to determine the time of year when pollution levels in Bengaluru City peaked. The Air Quality Index (AQI) was then calculated for all 12 monitoring stations using the AQI formula, and a forecasting model was developed to predict future AQI values for Bengaluru. The accuracy of the model was assessed by comparing the forecasted AQI values with the actual recorded values. In the final phase, Structural Health Monitoring was carried out in areas with high pollutant levels. Non-destructive testing methods, including the Rebound Hammer Test, Ultrasonic Pulse Test, Open Circuit Potential Test, and Carbonation Test, were employed to evaluate the structural health of both old and new buildings in these highly polluted regions. This comprehensive approach ensured a thorough understanding of the interplay between air quality and structural integrity in urban environments.

### Data acquisition and preprocessing

Ambient air quality data for six pollutants (SO_2_, NO_x_, PM_10_, PM_2.5_, NH_3_, and Lead) were collected from 12 Continuous Ambient Air Quality Monitoring Stations (CAAQMS) managed by the Karnataka State Pollution Control Board (KSPCB) and Central Pollution Control Board (CPCB) from 2013 to 2021. The stations are distributed across Bangalore’s North, South, East, West, and Central zones, as listed here:

- Export Promotion Industrial Park ITPL, White Field.
- AMCO Batteries, Mysore Road
- Rail Wheel Factory, Yelahanka
- Swan Silk Ltd., Peenya Ind. Area
- Victoria Hospital, K. R. Market
- Yeswanthapura Police Station
- Terri Office, Domlur
- Central Silk Board, Hosur Road
- Indhira Gandhi Children Health Care Centre, Jayanagar.
- Kazisonnenahalli
- Banasavadi Police Station, Kammanahalli
- Urban Eco Park, KSPCB Office, Peenya

The instrumentation used for data collection included Beta Attenuation Monitors (BAM) for PM_10_ and PM_2.5_, chemiluminescence detectors (Thermo Fisher Scientific Model 42i) for NO_x_, Ultraviolet Fluorescence (UVF) analysers (Ecotech Serinus 30) for SO_2_, and passive diffusion tubes with ion chromatography (Dionex ICS-5000) and X-ray fluorescence (XRF) for NH_3_ and Lead, respectively. Data preprocessing involved addressing missing data, which accounted for less than 5% of the total dataset. Gaps were filled using linear interpolation after verifying station logs for maintenance periods. Outliers, defined as values beyond ± 3 standard deviations from monthly means, were excluded, affecting less than 1% of the data. Hourly data were aggregated into daily averages to align with CPCB’s Air Quality Index (AQI) calculation framework.

### Comparative analysis of pollutants

A comparative analysis of pollutant concentrations across the 12 monitoring stations was conducted using ANOVA (α = 0.05) implemented in Python’s SciPy library. Stations with consistently high levels of PM_10_ and PM_2.5_, such as Central Silk Board and Peenya, were identified through post-hoc Tukey tests. To identify factors contributing to elevated pollution levels, field surveys were conducted monthly at each station (three surveys per station) to document traffic density, construction activity, and industrial emissions. Using Geographic Information System (GIS) mapping through QGIS, the proximity to highways, industries, and construction zones was quantified within a 500-m buffer radius. Additionally, interviews were conducted with 15 officials from the Karnataka State Pollution Control Board (KSPCB) to cross-verify pollution sources and validate the findings.

### Seasonal variation analysis

Seasonal variations in pollutant concentrations were analysed by defining three seasons: Summer (March–May), Monsoon (June–September), and Winter (October–February). Seasonal means were compared using the Kruskal–Wallis test, suitable for non-normal data distributions, followed by Dunn’s post-hoc analysis for pairwise comparisons. Violin plots, generated using Matplotlib, visualised the distributions of PM_10_, PM_2.5_, and NO_x_ across the three seasons. This analysis revealed that PM_10_ and PM_2.5_ concentrations were highest during summer and winter, while NO_x_ levels remained consistently low across all seasons.

### AQI calculation and forecasting model

The Air Quality Index (AQI) was calculated using CPCB’s breakpoint table (which has been tabulated along with colour codes based on guidelines from the U.S. EPA and Indian CPCB, as depicted in Table 1) and the sub-index formula as shown in Eq. (1):

1 $$I_{p} = \left[ {\frac{HI - LO}{{BP_{HI} - BP_{LO} }} \times \left( {C_{p} - BP_{LO} } \right)} \right] + LO$$

where ${C}_{p}$ represents the measured concentration, ${BP}_{HI}$ and ${BP}_{LO}$ are the breakpoint ranges, and $HI$ and $LO$ are the AQI category bounds. The highest sub-index value for each station was selected as the actual AQI value.

**Table 1:** Indian CPCB AQI levels. *Source* National Ambient Air Quality Index by Central Pollution Control Board.

Range	Category	Possible health impacts
0–50	Good	Minimum impact
51–100	Satisfactory	Minor breathing discomfort to sensitive people
101–200	Moderate	May cause breathing discomfort to people with lung diseases such as asthma and discomfort to people with heart disease in children and older adults
201–300	Poor	May cause breathing discomfort to people on prolonged exposure and discomfort to people with heart disease
301–400	Very poor	May cause respiratory illness in people on prolonged exposure. The effect may be more pronounced in people with lung and heart disease
> 401	Severe	May cause respiratory effects even in healthy people and serious health effects on people with lung/heart disease

A forecasting model was developed using polynomial regression (degree = 2) implemented in R with the caret package. The dataset was split into 80% (2013–2019) for training and 20% (2020–2021) for testing. The model achieved an ${R}^{2}$ value of 0.93, with a root mean square error (RMSE) of 18.2 and a mean absolute error (MAE) of 14.5, indicating strong predictive accuracy.

### Structural health monitoring

Structural health monitoring was conducted on buildings located in three high-pollution zones: Central Silk Board, Peenya Industry, and K.R. Market. Both old (18 years) and new (1 year) buildings were assessed using Non-destructive Testing (NDT) techniques. The *Rebound Hammer Test* was performed using a Proceq SilverSchmidt (ASTM C805). Twenty readings were taken per column, and outliers (± 2 standard deviations) were excluded before calculating the mean compressive strength. The *Ultrasonic Pulse Velocity (UPV) Test* was conducted using a PUNDIT Lab + with 54 kHz transducers (ASTM C597). Grease-coupled transducers measured pulse velocity, and results were categorised based on concrete quality using predefined thresholds. The *Open Circuit Potential (OCP) Test* involved scanning reinforcement bars at 10 cm intervals using a Saturated Calomel Electrode (SCE) (ASTM C876). Corrosion risk was classified based on measured potential values, with thresholds defined in Table 9. The *Carbonation Test* was performed by splitting concrete cores and spraying them with 1% phenolphthalein. The depth of carbonation was measured using digital callipers (Mitutoyo, 0.01 mm precision), and results were used to assess the likelihood of steel reinforcement corrosion.

## Results and analysis

The results of the study present the results obtained from a comparison between different types of emissions from all stations, a study on seasonal variations, the development of a forecasting regression model for finding out AQI in Bangalore, and Structural Health Monitoring on buildings located in highly polluted areas.

### Comparison of different types of emissions from all the stations located in Bengaluru

**Fig. 2:**
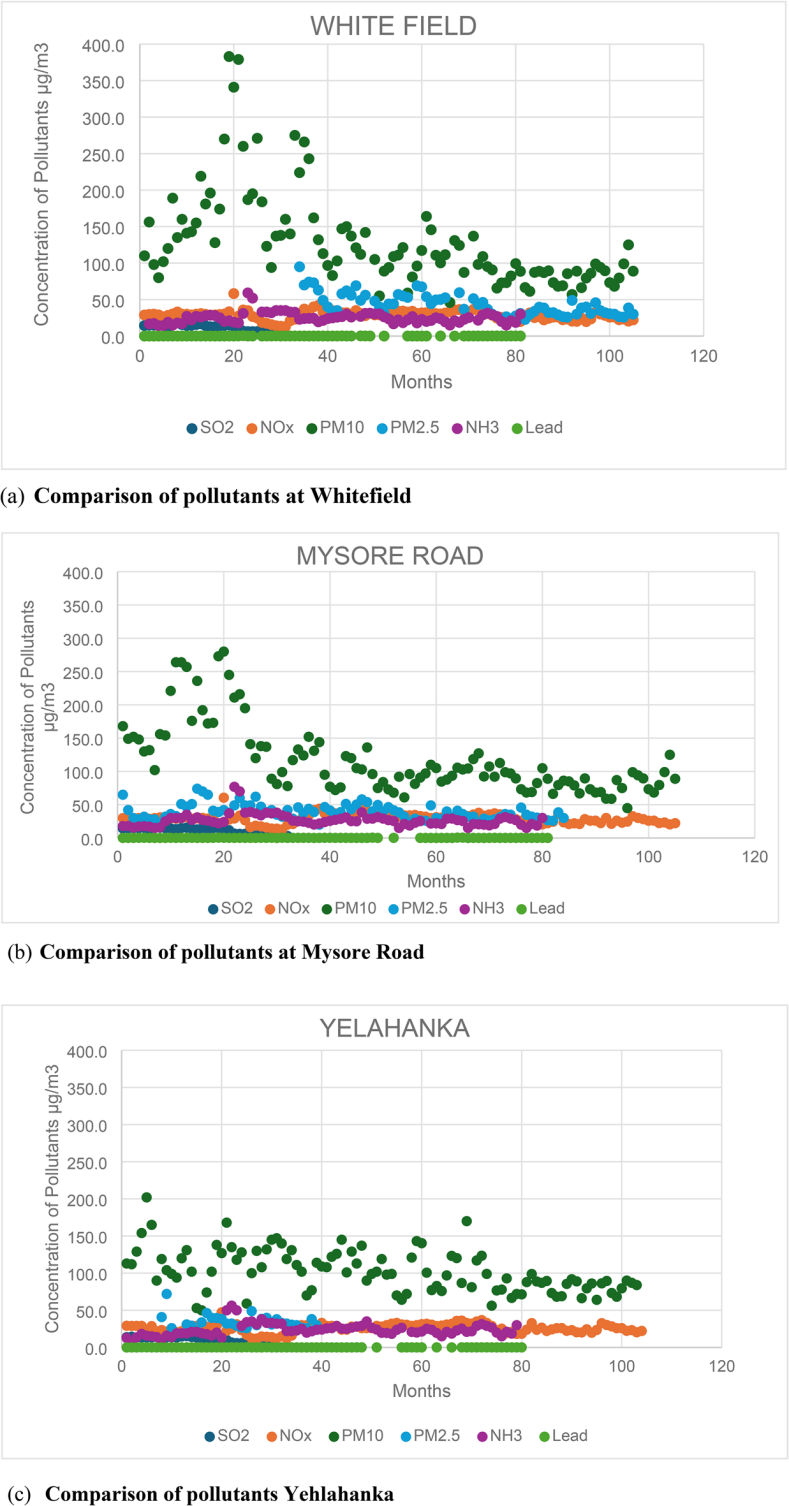

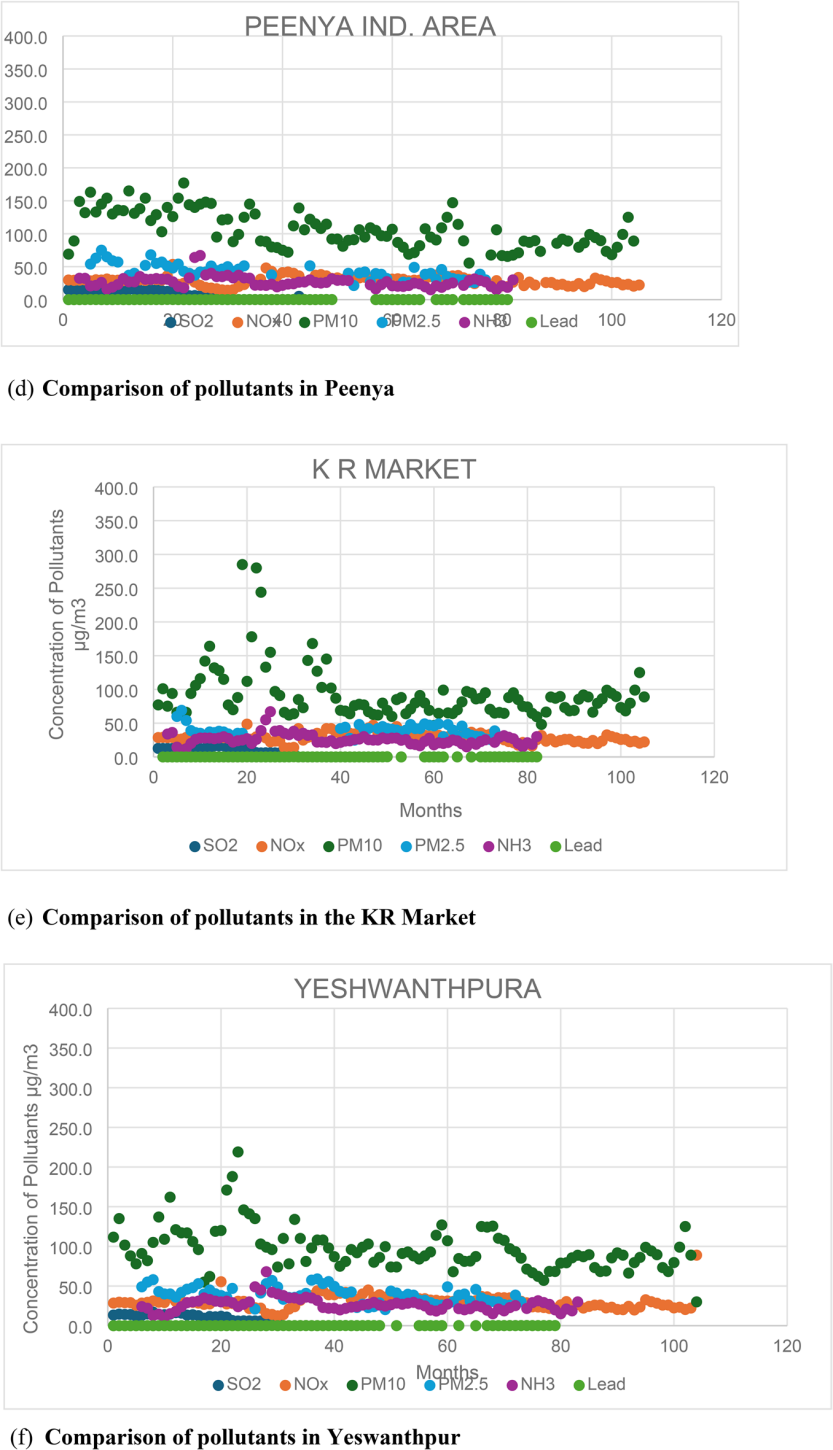

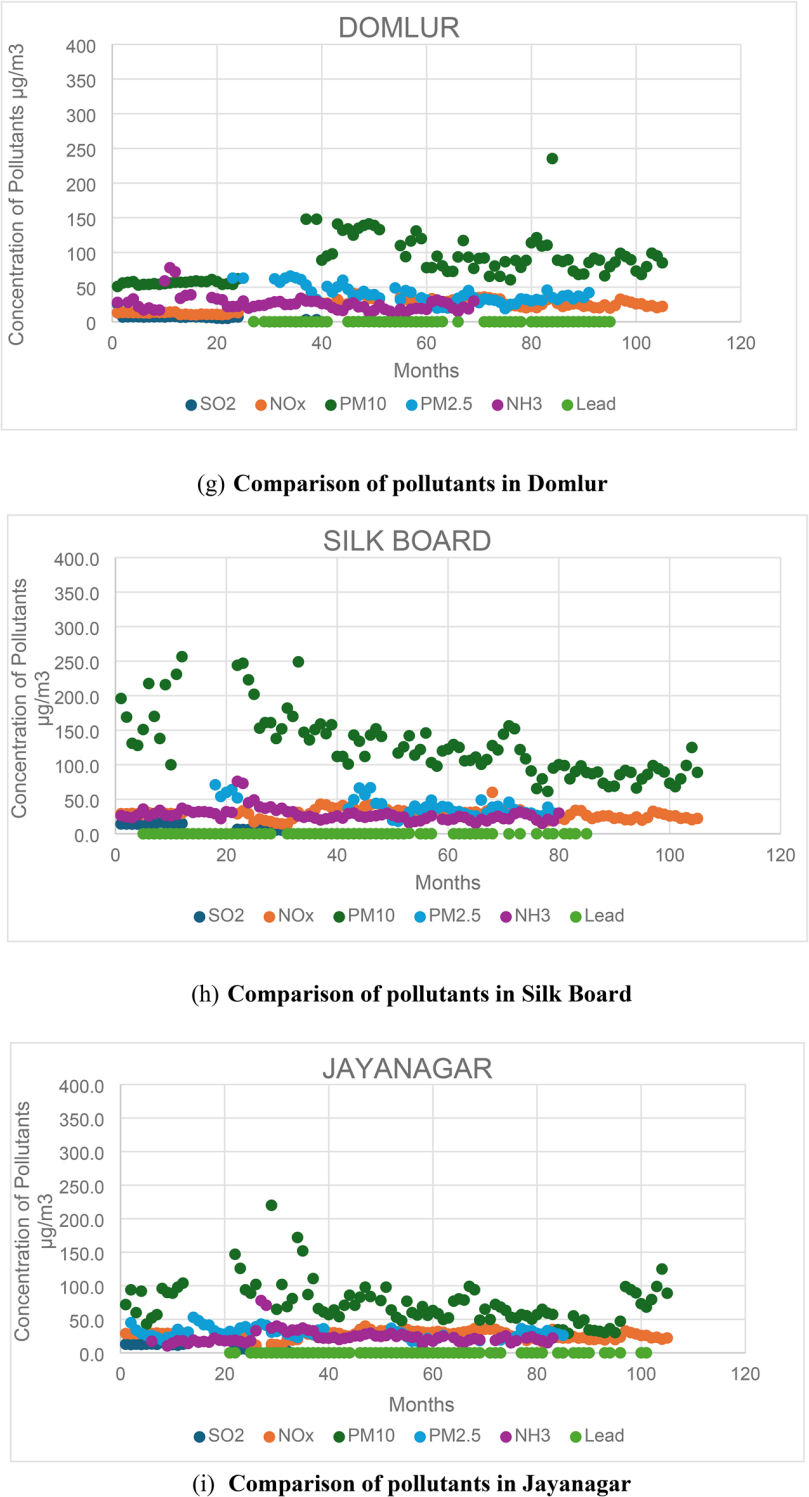

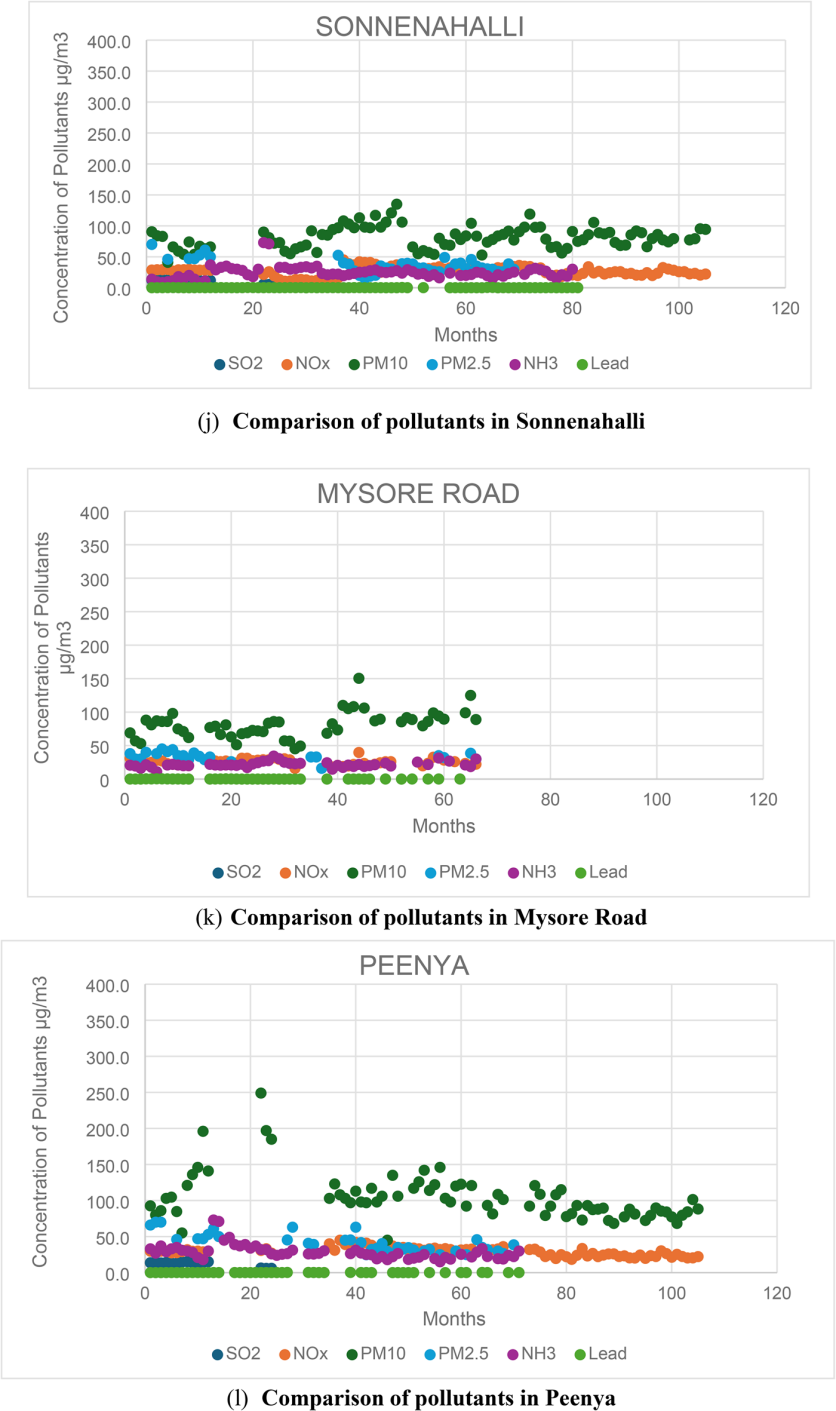
Comparison of pollutants at all the 12 stations.

A comparison is conducted across all six emissions (SO2, NOx, PM10, PM2.5, NH3, and Lead) from the 12 stations to identify the pollutants responsible for high pollution levels. By analysing these pollutants, the stations with the highest pollution levels are identified, along with the factors influencing these pollutants. Figure 2 illustrates the comparison of all six emissions across the 12 stations. Additionally, the total number of samples exceeding the maximum NAAQS (National Ambient Air Quality Standards) is determined from all 12 stations.

Based on the findings, it is evident that PM10 is the primary pollutant responsible for pollution, with approximately 99% of total samples from all 12 stations surpassing the maximum limit of 60 µs/m^3^. PM2.5 follows closely, with approximately 85% of the total samples exceeding the permissible limit of 40 µg/m^3^. In contrast, NOx is the least significant pollutant, with fewer than 5% of the total samples surpassing the limit of 40 µg/m^3^. Among the six pollutants analysed, PM10, PM2.5, and NOx stand out as the primary contributors to pollution in Bengaluru City.

### Factors influencing PM10, PM2.5 and NOx

The emissions in Bengaluru City are influenced by various factors, including vehicle exhaust, construction dust, on-road dust, industrial activities, IT companies, population density, vehicular traffic, proximity to national highways, metro and flyover construction, waste burning, transportation activities, fossil fuel combustion, microbial decaying processes, demolition activities, and indoor air pollution. These factors contribute to the generation of pollutants such as PM10, PM2.5, NOx, SO2, NH3, and Lead. Table 2 highlights that stations like Central Silk Board, Peenya Industry, and K. R. Market have notably high emissions of PM10, PM2.5, and NOx. The factors identified in these areas are primarily responsible for the elevated pollution levels observed for these pollutants.

**Table 2:** Factors influencing the emissions.

Factors influencing NOx, PM10 and PM2.5		Stations											
		a	b	c	d	e	f	g	h	i	j	k	l
1	Vehicle exhaust	**–**		**–**	**–**	**–**	**–**	**–**	**–**	**–**	**–**	**–**	**–**
2	Construction dust	**–**	**–**		**–**	**–**	**–**	**–**	**–**	**–**	**–**	**–**	**–**
3	On-road dust	**–**	**–**	**–**	**–**	**–**	**–**	**–**	**–**	**–**	**–**	**–**	**–**
4	Industries				**–**				**–**				
5	IT company		**–**	**–**			**–**		**–**				
6	Population	**–**	**–**	**–**	**–**	**–**	**–**	**–**	**–**	**–**	**–**	**–**	**–**
7	Vehicular traffic	**–**	**–**	**–**	**–**	**–**	**–**	**–**	**–**	**–**			**–**
8	National highway		**–**	**–**	**–**				**–**				**–**
9	Metro construction	**–**		**–**	**–**	**–**	**–**		**–**				**–**
10	Fly over construction		**–**		**–**				**–**				
11	Burning of waste			**–**	**–**	**–**	**–**	**–**	**–**	**–**			
12	Transportation	**–**	**–**	**–**	**–**	**–**	**–**	**–**	**–**	**–**	**–**	**–**	**–**
13	Burning of fossil fuels	**–**	**–**		**–**	**–**		**–**	**–**				
14	Microbial decaying process				**–**	**–**			**–**				
15	Demolition	**–**			**–**	**–**			**–**				
16	Indoor air pollution	**–**	**–**		**–**	**–**		**–**	**–**	**–**	**–**	**–**	

### Study on seasonal variation of PM10, PM2.5 and NOx emissions

**Fig. 3:**
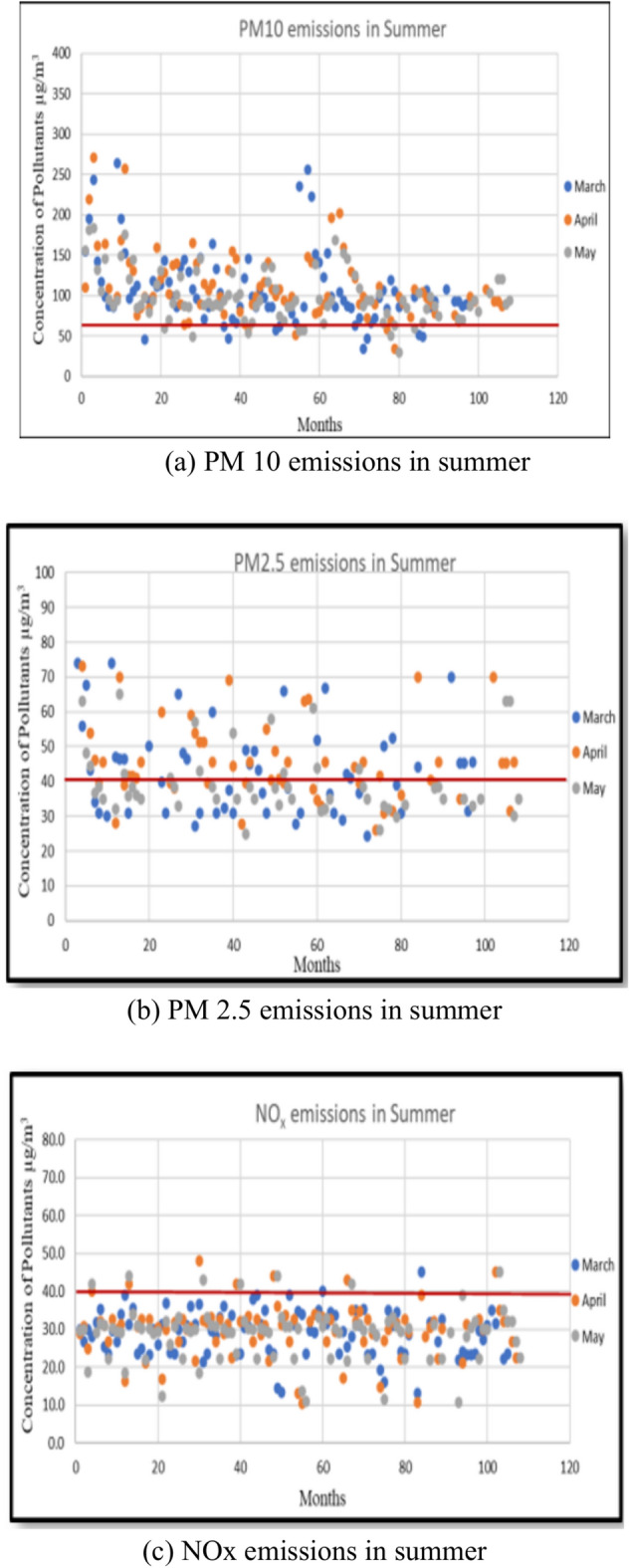
PM 10, PM 2.5, and NOx emissions during summer.

**Fig. 4:**
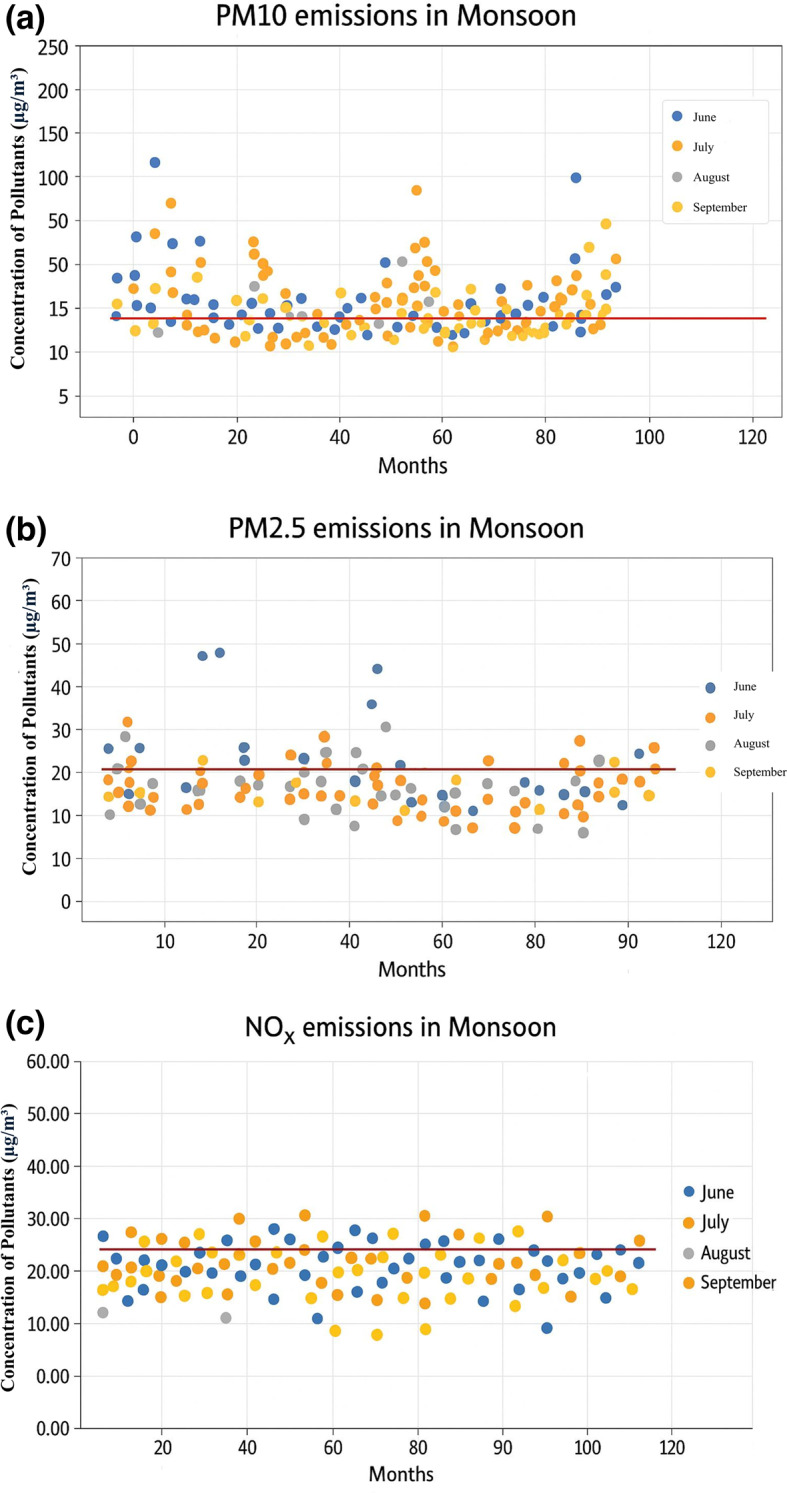
PM 10, PM 2.5, and NOx emissions in monsoon.

**Fig. 5:**
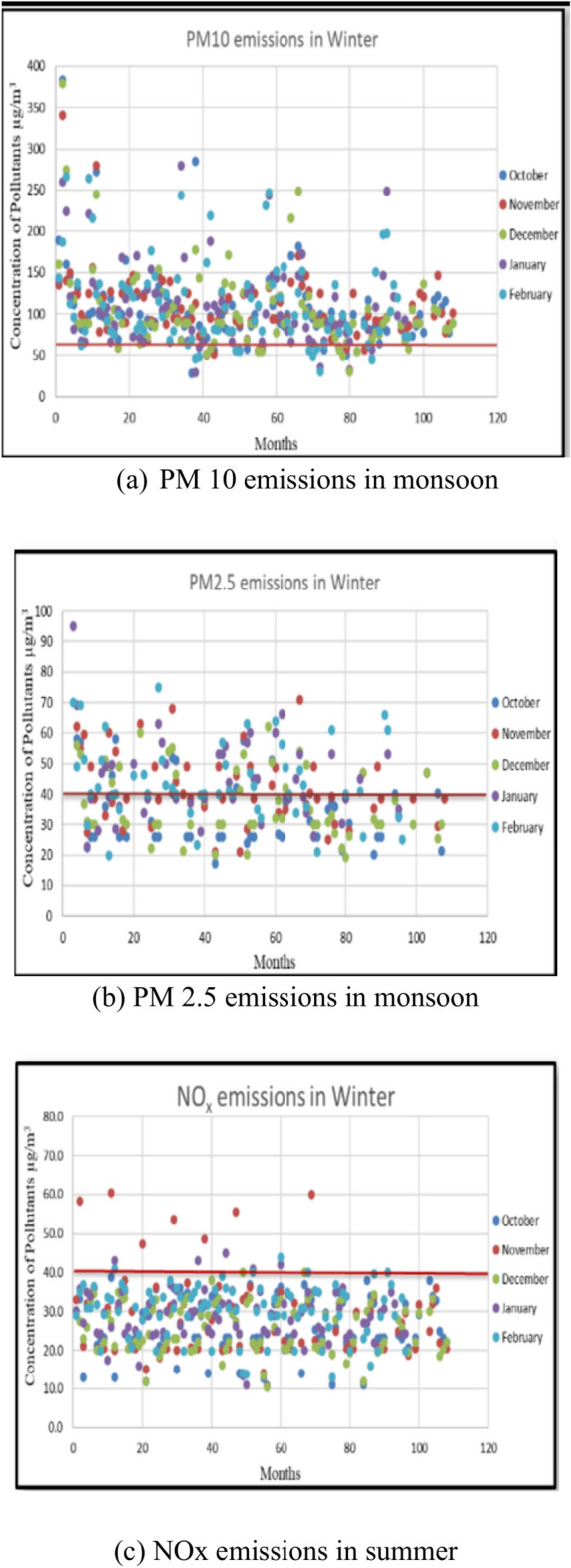
PM 10, PM 2.5, and NOx emissions in monsoon.

A study was conducted to ascertain which season experiences heightened emissions. Separate graphs were plotted for PM10, PM2.5, and NOx, disregarding station locations. From each graph, the number of samples surpassing the limit specified by NAAQS was determined. The Summer, Monsoon, and Winter seasons were compared to identify the season with elevated emission-causing pollutants. Figures 3, 4, and 5 illustrate the seasonal variability of emissions from all 12 stations. The study on the seasonal fluctuation of PM emissions reveals that during summer, approximately 95% of samples exceed the limit, while during winter, this figure stands at 92%. Consequently, the concentration of PM10 emissions is notably high in summer compared to the monsoon, with nearly 88% of samples surpassing the limit. Similarly, PM2.5 concentrations reach their highest levels during summer, with 60% of samples exceeding the permissible limit. This is followed by a moderate rise in winter, where around 50% of samples surpass the limit, and a significant decline during the monsoon, with fewer than 16% exceeding the limit. In contrast, NOx emissions remain consistently low across all three seasons. As a result, PM concentrations are highest in summer and winter and lowest during the monsoon. The findings suggest that both summer and winter seasons experience higher pollution levels compared to the monsoon season.

Srimuruganandam and Nagendra^4^ identified seasonal variations, demonstrating that PM mass concentrations peak during the post-monsoon period, followed by winter and then summer (observed in Chennai City). Meanwhile, Srimuruganandam and Nagendra^6,7^ found that PM emissions concentrations were highest during the monsoon and winter seasons and lowest during summer. Similarly, Srimuruganandam and Nagendra^8^ reported that elevated PM (PM10 and PM2.5) concentrations occurred during summer and monsoon seasons. On the other hand, Suman et al.^14^ revealed that in Kanpur City, high PM emissions concentrations were recorded during summer and winter, with lower levels during the monsoon season. Notably, while significant research has been conducted in Chennai City, where high emission concentrations are observed during post-monsoon and winter seasons, the results obtained in this study differ. Conversely, the findings in Kanpur City align closely with the seasonal variations observed in this study, with high emissions concentrations during summer and winter and lower concentrations during the monsoon season.

### Developing of forecasting model for finding out AQI in Bangalore

#### AQI calculations

To develop a model using AQI values, the first step involves calculating the AQI values for all monitoring stations using the monthly average of AQI in µg/m^3^. The formula used to compute the AQI value is provided in Eq. (1).

1 $$I_{p} = \left[ {\frac{HI - LO}{{BP_{HI} - BP_{LO} }} \times \left( {C_{p} - BP_{LO} } \right)} \right] + LO$$

where Ip: Sub-Index Value (no unit), Hi: Highest Indian AQI value , Lo: Lowest Indian AQI value, BPHi: Higher Indian range of AQI µs/m^3^, BPLo: Lower Indian Range of AQI µs/m^3^, Cp: AQI value µs/m^3^.

Out of the total 3000 samples collected from all 12 stations, those surpassing the NAAQS limits are identified, and a sub-index is calculated for each station. The highest sub-index value, regardless of station or pollutant, is selected and tabulated as the Actual AQI value. For the actual AQI value, the predominant pollutant is identified and listed. Additionally, colour coding based on Indian CPCB AQI levels (referencing Table 1) is applied to the Actual AQI values. This information is presented in Table 3.

**Table 3:** Calculation of Actual AQI value.

Year 2013–2021	Sub-index value from all the 12 stations												AQI	Prominent Pollutant
1	230	341	126	136	235	148	61	0	0	0	0	0		PM10
2	128	225	132	132	137	128	0	199	180	92	0	0	225	PM10
3	132	217	130	126	137	106	132	140	108	132	88	132	217	PM2.5
4	96	128	139	110	130	100	128	131	98	80	79	131	139	PM2.5
5	118	143	127	105	97	117	112	120	99	103	86	114	143	PM10
6	104	125	139	110	66	93	114	115	68	98	107	114	139	PM2.5
7	89	95	92	92	92	92	92	92	55	92	92	89	95	PM10
8	117	117	90	117	117	117	99	117	117	95	117	101	117	PM10

#### Forecasting model to determined

**Fig. 6:**
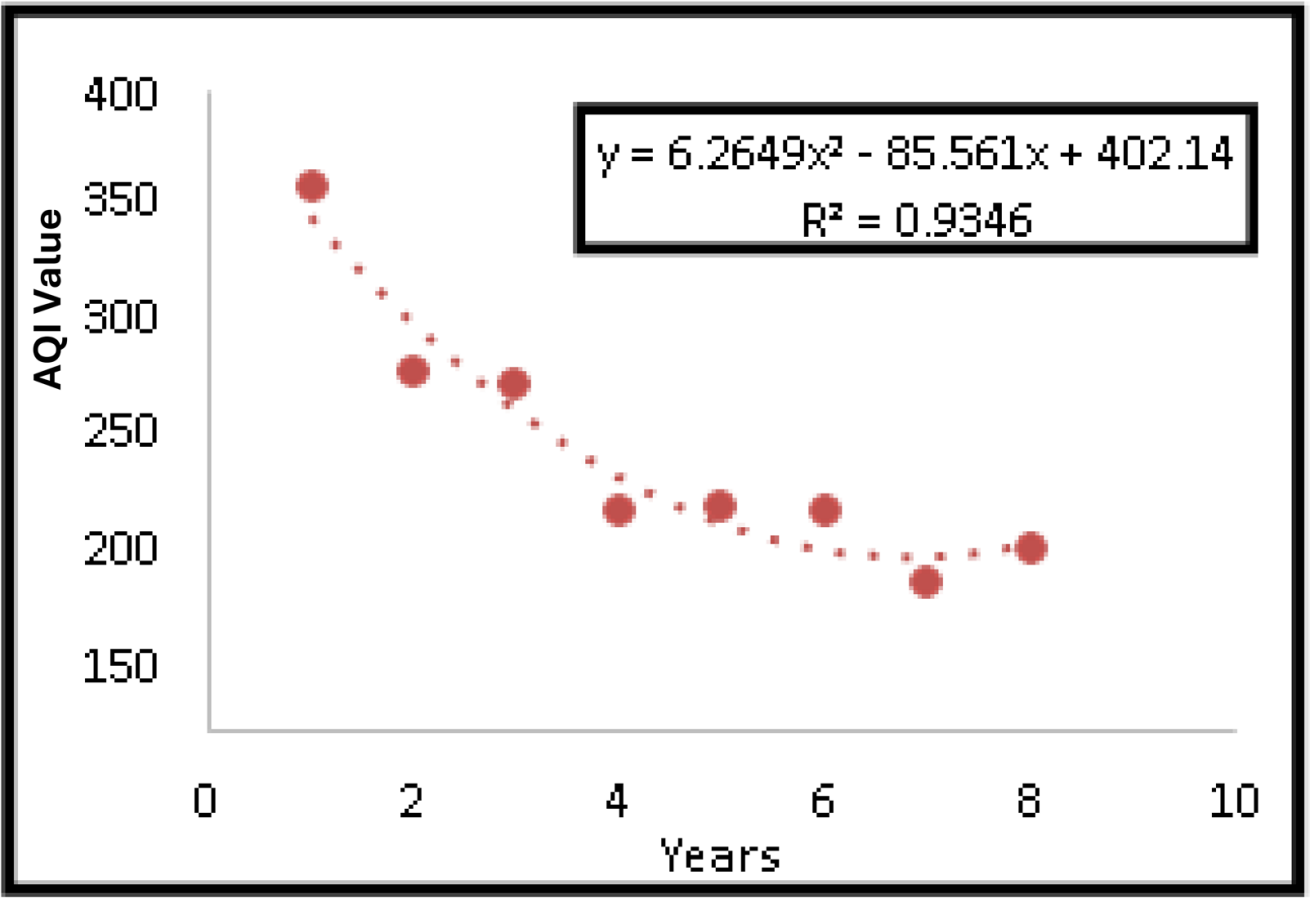
Forecasting model to predict AQI.

A forecasting model is developed between Year v/s actual AQI value. Figure 6 presents the forecasting model of the determined AQI value. From the forecasting model, a linear regression equation is obtained and is shown as Eq. (2)

2 $${\text{y}} = {6}.{\text{2649x2}} - {85}.{\text{561x}} + {4}0{2}.{14} \ldots$$

where x = years (from 2013–2021) , y = actual AQI value, R^2^ = coefficient of determination.

The obtained R^2^ = 0.93 indicates that 93% of the Actual AQI value fits the collected data. Hence, the determined work is highly correlated.

#### Comparison of predicted and actual AQI value

Table 4 displays the Predicted and Actual AQI values, with the predicted values derived from the linear regression equation developed during the forecasting model’s creation. To assess the regression model, a comparison is made between the expected and actual AQI values. The evaluation of the regression model reveals a variation of approximately 10% between the predicted and actual AQI values.

**Table 4:** Predicted AQI value and Actual AQI value.

Predicted value of AQI	Actual value of AQI
323	341
256	225
202	217
160	139
131	143
114	139
110	95
119	117

### Structural health monitoring

For Structural Health Monitoring of buildings located in highly polluted areas, NDT (Non-destructive Testing) is to assess their strength and durability, and detect any potential defects. The aim is to provide precise, quantitative data on the building’s current condition and recognise signs of ageing.

The following are the NDT tests conducted on buildings (Old and New).

- Rebound Hammer Test
- Ultrasonic Pulse Velocity Test
- Open Circuit Potential Test
- Carbonation Test

#### Rebound hammer test

The rebound hammer test is performed on both old and new buildings situated in highly polluted areas, specifically at Central Silk Board, Peenya Industry, and K. R. Market. This test aims to assess the structural strength of the buildings, considering their respective lifespans of 18 years (for the old building) and 1 year (for the new building). Column members are selected in both buildings for the rebound hammer test, wherein the hammer is applied perpendicular to the column surface to calculate compressive strength. Table 5 displays the compressive strength results of the old and new buildings at the three locations. A comparison is drawn between old and new buildings to enhance understanding of the study. For instance, at Central Silk Board, the compressive strength of the old building is 21 N/mm^2^, whereas the new building registers 32 N/mm^2^. This discrepancy indicates lower strength in the old building, likely due to deterioration, mainly attributed to air pollution stemming from construction activity and vehicular traffic. Conversely, the old buildings at the other two stations exhibit no concrete decline when compared to the old building at Central Silk Board.

**Table 5:** Compressive strength of old and new buildings.

Sl. no	Stations	Compressive strength N/mm^2^	
		Old building	New building
1	Central silk board	21	32
2	Peenya industry	30	28
3	K. R. market	32	27

#### Ultrasonic pulse velocity test

The subsequent NDT test performed was the ultrasonic pulse velocity test. For this test, one old building and one new building, previously selected for the rebound hammer test, were chosen again. The structural element considered for the ultrasonic pulse velocity test was also a column. Results obtained from the UPV test are presented in Table 6, which assesses the concrete quality of the buildings. Analysis of Table 6 reveals that, at Central Silk Board, the concrete quality of the old building is notably poorer compared to the other two old buildings (Peenya Industry and K. R. Market), which demonstrate medium quality. Conversely, the concrete quality of the new buildings is rated as good and excellent. Each building’s concrete quality is graded according to the specifications outlined in Table 7

**Table 6:** Ultrasonic Pulse Velocity of an old building and a new building.

Sl. no	Stations	Old building		New building	
		Pulse velocity m/s	Concrete quality	Pulse velocity m/s	Concrete quality
1	Central Silk Board	2875	Poor	3325	Good
2	Peenya Industry	3202	Medium	3711	Good
3	K. R. Market	3450	Medium	4713	Excellent

**Table 7:** Suggested pulse velocity for concrete quality grading.

Sl. no	Pulse velocity (m/s)	Concrete quality grading
1	> 4500	Excellent
2	3500–4500	Good
3	3000–3500	Medium
4	< 3500	Poor

#### Open circuit potential test

In this test, the chosen structural element under examination is concrete, with reinforcement bars secured at the ends. Results obtained from the open circuit potential test conducted on both old and new buildings at Central Silk Board, Peenya Industry, and K. R. Market are outlined in Table 8. This test aims to evaluate the occurrence of corrosion in these buildings and determine the percentage of corrosion present. The data in Table 8 clearly shows that the old building at Central Silk Board displays significant corrosion compared to the other two old buildings, where corrosion levels are comparatively lower. To accurately determine the exact percentage of corrosion in the remaining two old buildings, additional tests are carried out. Conversely, in new buildings, the likelihood of corrosion is observed to be below 10%. Table 8 provides insights into the relationship between Potential values and the probability of corrosion occurrence. Remarks for each building are determined using the guidelines provided in Table 9.

**Table 8:** Open circuit potential of old and new buildings.

Sl. no	Stations	Old building		New building	
		OCP mV	Remarks	OCP mV	Remarks
1	Central silk board	− 428	High probability of > 90% of corrosion	− 217	High probability of < 10% corrosion
2	Peenya industry	− 345	Uncertainty of corrosion	− 178	High probability of < 10% corrosion
3	K. R. market	− 330	Uncertainty of corrosion	− 155	High probability of < 10% corrosion

**Table 9:** Suggested corrosion probability for measured potential values.

Measured potential (mV)	Probability of steel corrosion
> − 200	Less than 10%
− 200 to − 350	Uncertain
< − 350	More than 90%

#### Carbonation test

In this test, the chosen structural element is a column. The methodology briefly outlines the procedure for conducting this test. Results obtained from the Carbonation Test are presented in Table 10. In this test, the depth of carbonation serves as an indicator of steel reinforcement corrosion and identifies crack formation in the buildings. As the depth of carbonation increases, the pH level decreases, consequently reducing the alkalinity in the concrete. In concrete of good quality, the pH value should exceed 12. A pH value below 7 indicates a high likelihood of corrosion in the building.

**Table 10:** Depth of Carbonation of old and new buildings.

Sl. no	Stations	Old building		New building	
		Depth of carbonation mm	Remarks	Depth of carbonation mm	Remarks
1	Central Silk Board	19	Cracks Formation and corrosion of steel (pH < 7)	2	Carbonation should not reach reinforcement level during the lifetime of the RCC structure. (pH > 12)
2	Peenya Industry	8		2	
3	K. R. Market	5		1	

## Discussion

Comparing the two cities, Bengaluru generally shows stronger correlations between temperature and SO2 across all seasons, while Chennai exhibits more variation in correlations between temperature, humidity, and pollutant levels across seasons. Additionally, Bengaluru’s correlations between moisture and pollutants are more consistent across seasons compared to Chennai. These differences could be attributed to various factors such as geographical location, urbanisation level, and local sources of pollution. The correlation patterns between PM_10_ and PM_2.5_ concentrations in Chennai and Bengaluru provide insights into air quality dynamics in both cities. In Chennai, both for summer and winter, there is a positive correlation between PM_10_ and PM_2.5_ concentrations, indicating that increases in PM_10_ levels are associated with increases in PM_2.5_ levels during these seasons. The strength of correlation is relatively high, with coefficients of determination (r^2^) of 0.76 and 0.74 for summer and winter, respectively. However, in monsoon, there is a moderate positive correlation between PM_10_ and PM_2.5_ concentrations during the monsoon season, with an r^2^ value of 0.54. This suggests a somewhat weaker association compared to summer and winter but still indicates a tendency for both pollutants to increase or decrease together during this season. In Bengaluru city, there is a moderate positive correlation between PM10 and PM2.5 concentrations across all seasons. The strength of correlation is consistent throughout the year, with r2 values of 0.48, 0.51, and 0.51 for summer, winter, and monsoon seasons, respectively. This suggests a stable relationship between PM10 and PM2.5 levels regardless of seasonal variations. Overall, both Chennai and Bengaluru exhibit positive correlations between PM_10_ and PM_2.5_ concentrations, indicating that changes in one pollutant are associated with changes in the other. However, Bengaluru shows a more consistent correlation pattern across all seasons compared to Chennai. These findings can inform air quality management strategies and help identify potential sources and factors influencing particulate pollution in both cities.

## Conclusions

The primary objective of this project is to assess and model PM emissions and their impact on buildings. Through a comparative analysis of various emissions, it is evident that PM10, PM2.5, and NOx are the pollutants contributing significantly to high pollution levels in Bangalore City. Specifically, monitoring stations such as Central Silk Board, Peenya Industry, and K. R. Market are identified as having elevated pollution levels compared to the other 12 stations across Bangalore City. Several factors contribute to these pollutants, including vehicle exhaust, construction dust, on-road dust, industrial activities, IT companies, population density, vehicular traffic, proximity to national highways, metro and flyover construction, waste burning, transportation emissions, fossil fuel combustion, microbial decay, demolition activities, and indoor air pollution.

The analysis of seasonal variation reveals that pollution concentrations are notably higher during the summer and winter seasons compared to the monsoon season. Consequently, a forecasting model is developed to predict the Air Quality Index (AQI) in Bangalore City. Through this model, a linear regression equation is derived, yielding a coefficient of correlation (R2) of 0.93. The high R2 value suggests a strong correlation between the determined AQI values from the collected data. Utilising the linear regression equation, the predicted AQI values are computed. To assess the efficacy of the regression model, a comparison is conducted between the expected and actual AQI values. This comparison reveals approximately a 10% variation between the predicted and actual AQI values, indicating the model’s reliability.

In the culmination of structural health monitoring conducted on both old and new buildings, it is evident that in high-polluted areas, building materials and structural elements are significantly more affected compared to those in low-polluted areas. The primary findings regarding old buildings indicate a decrease in strength, deterioration of concrete, corrosion of steel reinforcement, diminished quality of concrete, and an increase in the depth of carbonation. Conversely, in new buildings, the structural members exhibit good strength, the quality of concrete is rated as good to excellent, there is a low probability of corrosion, and the depth of carbonation is minimal. These conclusions highlight the adverse impact of pollution on building materials and structural integrity, particularly in older structures, while also emphasising the benefits of newer construction methods and materials in mitigating such effects.

## Data Availability

The datasets generated during and/or analysed during the current study are not publicly available due to institutional norms but are available from the corresponding author upon reasonable request.
